# New Insights into the Role of Metformin Effects on Serum Omentin-1 Levels in Acute Myocardial Infarction: Cross-Sectional Study

**DOI:** 10.1155/2015/283021

**Published:** 2015-11-23

**Authors:** Hayder M. Alkuraishy, Ali I. Al-Gareeb

**Affiliations:** Department of Clinical Pharmacology and Therapeutic, College of Medicine, Al-Mustansiriya University, P.O. Box 14132, Baghdad, Iraq

## Abstract

*Background*. Serum omentin-1 level was low in the most types of ischemic heart disease compared to normal subjects; it also dependently correlated with coronary heart disease; thus, omentin-1 is regarded as a novel biomarker in IHD.* Objective*. The aim of the present study was to establish the links between omentin-1 and acute myocardial infarction in metformin patients.* Subjects and Methods*. A cross-sectional study was performed on eighty-five patients with type II DM and acute MI. They are divided as follows: Group I, 62 patients with type II DM who received metformin prior to onset of acute MI; Group II, 23 patients with type II DM who did not receive metformin prior to onset of acute MI; and Group III, 30 normal healthy controls. Venous blood was drawn from each participant for determination of lipid profile, plasma omentin-1, cardiac troponin-I (cTn-I) and other routine tests.* Results*. Patients that presented with acute MI that received metformin show a significant difference in all biochemical parameters (*p* < 0.001); metformin increases serum omentin-1 level and decreases serum cardiac troponin-I level compared with control subjects and nonmetformin treated patients.* Conclusion*. Metformin pharmacotherapy increases omentin-1 serum levels and may be regarded as a potential agent in the prevention of the occurrences of acute MI in diabetic patients.

## 1. Introduction

Acute myocardial infarction is one of the main diseases leading to death worldwide and is caused by atherosclerosis and other metabolic syndromes that induce inflammatory and immunological reactions that are caused by adipokines like Visfatin [1]. Omentin-1 is secreted from omentum and visceral fat also, it is found in the heart, lung, placenta, and ovary [2], it produced protective roles in regulating inflammatory and immunological response to induction of insulin sensitivity and downregulation of tumor necrosis factor (TNF) [3], and it inhibits free radicals and superoxide formations that play an important role in the vascular inflammation and smooth muscle remodeling; so it is regarded as protective adipokine in IHD [4]. Moreover, omentin-1 levels are inversely correlated with obesity, body mass index, hemoglobin, cholesterol, and type 2 diabetes mellitus [5]. Serum omentin-1 level was low in the most types of ischemic heart disease compared to normal subjects [6], but serum omentin-1 is positively correlated with high density lipoprotein (HDL) that is independently correlated with IHD, and many studies showed a significant correlation between low omentin-1 serum level and progressions of IHD [7].

Omentin-1 produced significant vasodilatations through induction of endothelial nitric oxide (eNO) phosphorylation; thus, NO inhibitor will abolish the vasodilating effect of omentin-1 [8]; additionally, it inhibits C-reactive protein and nuclear factor kappa-light chain of immune cells; so, it prevents the associations for inflammation, angiogenesis, and pathogenesis of coronary artery disease [9].

Additionally, omentin-1 triggers endothelial cells adenosine monophosphate protein kinase (AMPK) leading to potent anti-inflammatory action which modulates visceral fat macrophage activity [10].

Metformin is one of the most prescribed agents as a first line drug for treatment of type II diabetes mellitus worldwide; it acts through inhibition of hepatic glucose production and intestinal glucose absorption; also, it accelerates glucose uptake and consumption [11]. Long term uses of metformin lead to cardiovascular protection for prevention of microvascular and macrovascular complications which mediate diabetic harmful effect on the cardiovascular system [12].

Therefore, the aims of the present study are to establish the links between omentin-1 and acute myocardial infarction in patients treated with metformin prior to the onset of acute MI.

## 2. Subjects and Methods

This cross-sectional study was performed on eighty-five patients with type II DM with the age of 49–66 years that were screened for acute myocardial infarction at the coronary care unit (CCU) in Al-Yarmook Teaching Hospital in cooperation with Clinical Pharmacology, College of Medicine, Al-Mustansiriyia University, from January to July, 2015; this study was approved by the Scientific Committee Review Board in the first of January 2015 (ethical committee permission number 159). All enrolled patients offered oral and written informed permission for this study and acquiescence to use their samples for prospect study. The patients were selected according to grading of New York Heart Association (NYHA) [13].

### 2.1. Study Design

Eighty-five patients with type II DM (51 males + 34 females) and acute MI were selected. They are divided as follows: Group I, 62 patients with type II DM (42 males + 20 females) who received metformin prior to onset of acute MI; Group II, 23 patients with type II DM (9 males + 14 females) who did not receive metformin prior to onset of acute MI; and Group III, 30 normal healthy controls (20 males + 10 females) who have not received any drugs.

### 2.2. Exclusion Criteria

Patients with any other systemic diseases other than type II DM and acute MI are excluded.

### 2.3. Sample Processing

10 mL of venous blood was drawn after overnight fasting for lipid profile and other routine tests, and 3 mL was transferred into EDTA anticoagulant tubes centrifuged at 2000 r/min; then, supernatant was stored in the refrigerator to be used for estimation of plasma omentin-1, cardiac troponin-I (cTn-I), and other routine investigations by ELISA Kit, while atherogenic index (AI) = log(TG/HDL), low risk was <0.11, intermediate risk was 0.11–0.21, and high risk was >0.21.

### 2.4. Estimation of Serum Omentin-1 Concentration

Omentin-1 concentration was determined by ELISA Kit (Wellbiotechnology, Changsha) pg/mL.

### 2.5. Estimation of Plasma Troponin-I Concentration

Troponin-I concentration was determined by ELISA Kit (Cardiac Troponin-I ELISA Kit Catalog number E-EL-R1253 pg./mL, Elabscience, China).

### 2.6. Statistical Analysis

SPSS 22.0 software was used for statistical analysis, when *p* < 0.01 was regarded as significant. The variables were presented as mean ± SD; differences in mean values among groups were compared by ANOVA. Correlations between serum concentrations of omentin-1 and other variables were done via Pearson correlation analysis.

## 3. Results

Patients with acute MI at a coronary care unit (CCU) presented with miscellaneous clinical and biochemical variables with complete preceding history (Table 1).

The patients that presented with acute MI that received metformin show a significant difference in all biochemical parameters (*p* < 0.001). Serum omentin-1 was 27.13 ± 1.55 pg/mL in control subjects and 25.43 ± 1.2 pg/mL in patients with acute MI that were previously treated with metformin prior to the occurrence of MI, while serum cardiac troponin l increased from 21.202 ± 0.636 pg/mL in normal healthy controls to 75.453 ± 5.62 pg/mL in the patients with acute MI that were previously treated with metformin prior to the occurrence of MI. Other biochemical parameters also showed the same difference (Table 2).

In the patients who presented with acute MI in the coronary care unit (CCU) that were not previously treated with metformin, there is a significant difference in all biochemical parameters (*p* < 0.0001). Regarding serum omentin-1 level, it decreased from 27.13 ± 1.55 pg/mL in healthy controls to 23.71 ± 1.161 pg/mL in patients with MI not previously treated with metformin, while serum cardiac troponin-I increased from 21.202 ± 0.636 pg/mL in healthy controls to 79.53 ± 3.60 pg/mL in patients with MI not previously treated with metformin (Table 3).

Furthermore, there is a significant difference in most of biochemical parameters regarding serum omentin-1 and cardiac troponin-I between the patients that received metformin and the patients that did not receive metformin prior to occurrences of acute MI (*p* < 0.01). Thus, metformin elevates omentin-1 from 23.71 ± 1.161 pg/mL to 25.43 ± 1.2 pg/mL and lower serum cardiac troponin-I from 79.53 ± 3.60 pg/mL to 75.453 ± 5.62 pg/mL significantly in patients who presented with acute MI (Table 4).

**Figure 1:**
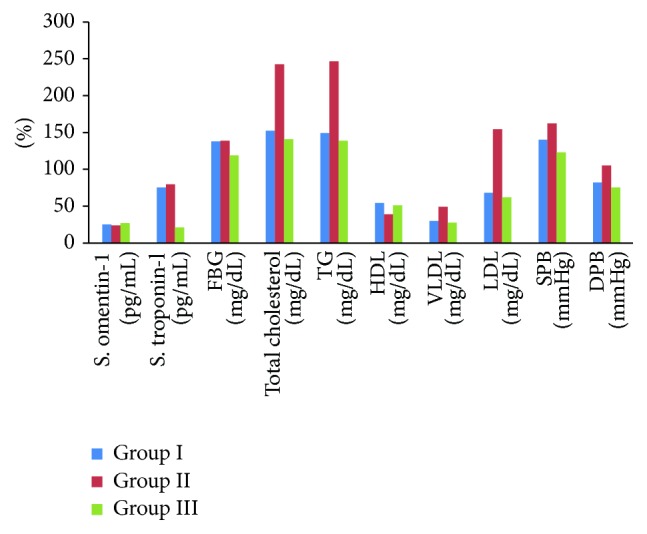
Biochemical parameter changes in acute MI regarding the previous metformin therapy in comparison with nonmetformin treated patients and healthy control subjects.

The results of the present study highlight the cardiovascular protective influence of pretreatment with metformin, manifested by ameliorating the acute MI associated biochemical changes, in patients with type 2 DM (Figure 1).

Regarding mean differences and 95% Confidence Interval of the difference (lower and upper limits) between control normal, healthy subjects and the patients with acute MI that either were previously treated with metformin or not, serum omentin-1 concentrations showed a high significant difference between those patients and normal controls (Table 5).

**Figure 2:**
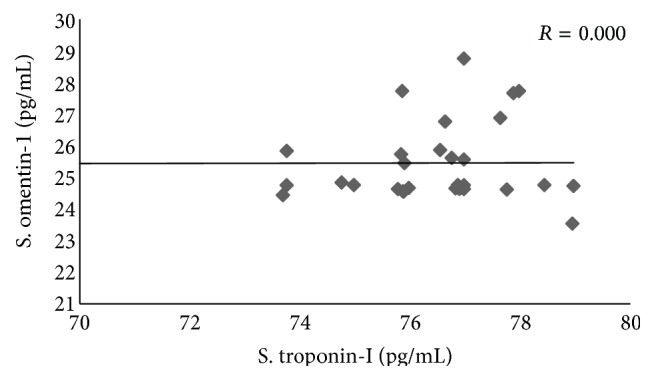
Correlation between serum omentin-1 and cardiac troponin-I in normal healthy control subjects.

Serum omentin-1 was not correlated with serum cardiac troponin-I in normal healthy subjects (*R* = 0.0) (Figure 2).

**Figure 3:**
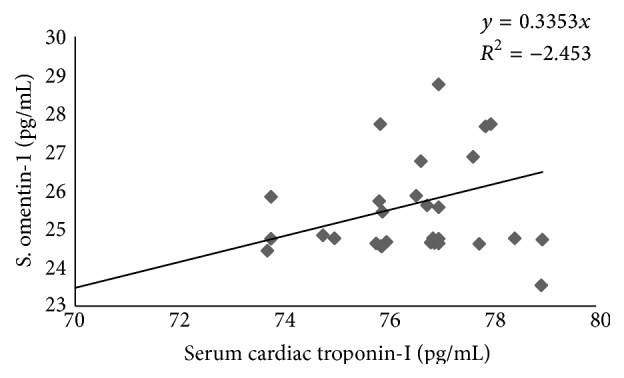
Correlation between serum omentin-1 and cardiac troponin-I in previously metformin treated patients with acute MI.

In previously metformin treated patients with acute MI, serum omentin-1 is significantly correlated with serum cardiac troponin-I (*R* = 1.56) (Figure 3).

**Figure 4:**
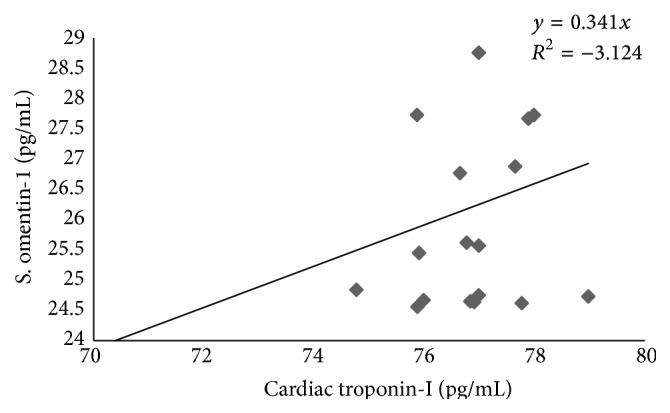
Correlation between serum omentin-1 and cardiac troponin-I in patients with acute MI not previously treated with metformin.

In the patients with acute MI that were not previously treated with metformin, serum omentin-1 is highly correlated with serum cardiac troponin-I (*R* = 1.76) (Figure 4).

**Figure 5:**
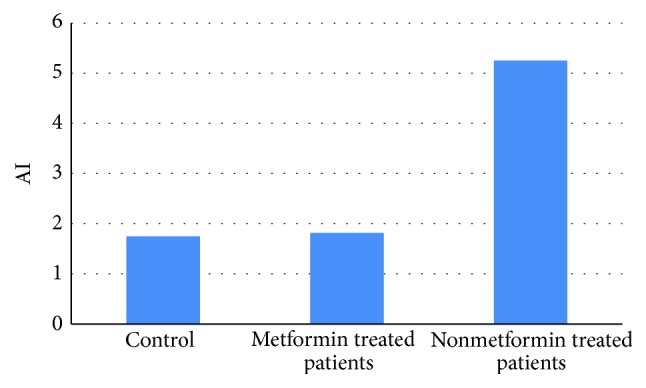
Differential atherogenic index levels in acute MI regarding metformin treatment.

Atherogenic index, which is mainly reflecting lipid profile effects, was high in nonmetformin treated patients (5.252) but it was low in metformin treated patients (1.814) and control healthy subjects (1.748) (Figure 5).

**Figure 6:**
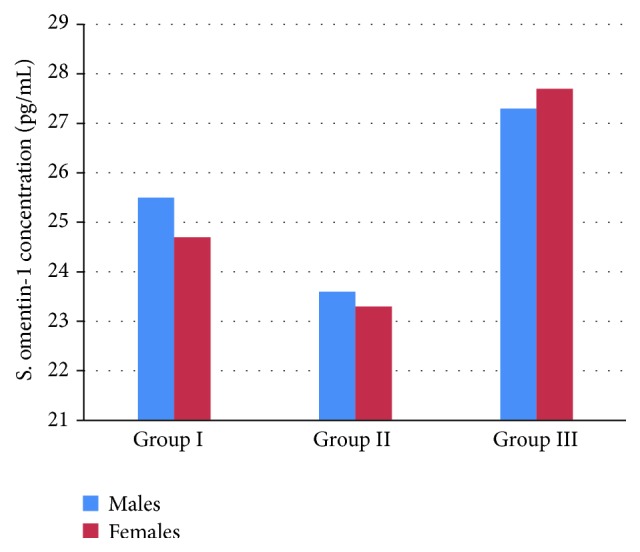
Gender differences in serum omentin-1 level in acute MI as compared with normal healthy group.

The interesting finding in the present study is the gender effects on the amplitude of changing in serum omentin-1 levels between healthy and diseased groups (Figure 6).

## 4. Discussion

The current study shows a significant change in the serum omentin-1 levels in patients who presented with acute MI at the CCU as compared with normal control subjects, since omentin-1 was expressed in visceral fat more than subcutaneous fat; thus, it decreased in the obesity and shows a negative correlation with BMI [14]. Low omentin-1 serum level was concerned with ischemic heart diseases [15]; also, omentin-1 induced NO production through activation of AMPK eNOS phosphorylation which suppressed p38-mediated e-selectin induction and so it improve vascular endothelial functions [16]. Therefore, omentin-1 effects on isolated rat blood vessels inhibit noradrenaline induced vasoconstriction and cause cardiac vasodilatations; also, omentin-1 reduced vascular endothelial dysfunction via amelioration of inflammatory process through inhibition of CRP, vascular endothelial growth factor (VEGF), NF-kB, and TNF [3]; these studies correspond with findings of present study which showed a significant reduction of omentin-1 serum concentrations in patients with acute MI in relation to BMI.

Thus, omentin-1 is regarded as protective adipokine in IHD and metabolic syndrome via inhibition of superoxide and free radical formations [17].

Moreover, findings of present study showed a considerable elevation in lipid profile and diminution in serum HDL level in relation to reduction of serum omentin-1 concentration.

Omentin-1 plays a potential role in lipid metabolism and insulin sensitivity thus, when omentin-1 decreased leading to insulin resistance and hyperinsulinemia which* per se* encourage hepatic triglyceride (TG) synthesis which blocks conversion of very low density lipoprotein (VLDL) into high density lipoprotein (HDL), this leads to high VLDL and low HDL which are concerned with progression of atherosclerosis and IHD; thus, omentin-1 is a protective factor in IHD [18, 19].

Regarding gender differences in omentin-1 serum level, female fat mass is higher than male fat mass, and because omentin-1 is negatively correlated with adiposity, since omentin-1 serum level is higher in male than female due to negative correlation between omentin-1 and ß-estradiol [20], this corresponds with our findings that showed a higher serum omentin-1 levels in males than females in patients with acute MI regardless of previous pharmacotherapy.

Moreover, a study showed that serum omentin-1 level did not differ significantly between males and females, and males with metabolic syndrome had 20% reduction in plasma omentin-1 as compared with females having the metabolic syndrome [21, 22].

The present study also showed that serum omentin-1 might predict cardiac measures in patients with acute MI.

Several adipokines and omentin-1 have beneficial roles, but the precise mechanism of these adipokines is still indistinct; also, low omentin-1 serum concentration may predict the occurrence of IHD [23]; in addition, the present study showed that serum omentin-1 levels positively correlated with cardiac troponin-I and this correlation may be of significance concerning peripheral biochemical disorders with cardiac ischemia.

Serum omentin-1 level was not correlated with BNP in heart failure, suggesting a different path-physiological progression of heart failure [24], but in IHD serum omentin-1 level is negatively correlated with cardiac troponin-I and IL-6, since both omentin-1 and IL-6 are produced by stromal vascular cells of fatty tissue with paracrine effect; so IL-6 may inhibit omentin-1 production, but cardiac troponin-I reflects cardiomyocyte damage that increased coincidentally with IL-6 [25].

Therefore, omentin-1 represents an optimistic biomarker for a prognosis of ischemic myocardial damage, when serum omentin-1 was low and high cardiac troponin-I indicates a poor prognosis, while normal or elevated omentin-1 serum level in IHD regardless of cardiac troponin-I indicates a good prognosis [26].

Metformin accounted for improving lipid profiles via decreasing plasma, serum levels of total cholesterol, triglyceride, LDL, VLDL, and increase in the HDL level [27]. Additionally, metformin was shown to lessen coagulation and accelerate fibrinolysis through inhibition of plasminogen activator factor inhibitor [28]; also, it reduced platelet activation and aggregation in the atherogenic vascular site [29].

Moreover, metformin was reported to prevent hyperglycemia induced cardiovascular damage through oxidative stress pathway since chronic hyperglycemia in DM activates protein kinase C (PKC) which leads to increases in the endothelial permeability, neutrophil activation, and cytokine stimulation, with all of these leading to vascular and cardiac complications of DM, therefore, metformin, leading to significant cardiovascular protection in diabetic patients through modulation of PKC [30, 31]. Long term effects of metformin lead to fatty acid oxidation stimulation and inhibition of PKC-NAD pathway which* per se* prevent vascular oxidative damage and inhibition of* de novo* diacylglycerol biosynthesis [32].

The pleiotropic effects of metformin are anti-ischemic activity, attenuation in ventricular postischemic dysfunction, raising tolerance to ischemia, and improving coronary blood flows leading to significant reduction in the incidence of MI [33].

So the present study confirmed the cardioprotective actions of metformin which might mediated, at least in part, via rising of omentin-1 serum level.

The cardioprotective mechanism of metformin was through activation of AMPK (which increases tolerance to ischemia), stimulation of endothelial NO synthase (which protects against ischemic-reperfusion), and anti-inflammatory effects via inhibition of IL-8, IL-6, IL-1, and TNF [34, 35].

Therefore, metformin increases serum omentin-1 levels which reflect improvement in insulin sensitivity and BMI; also, metformin is regarded as anti-inflammatory agent through modulation of certain cytokines that prevents the deleterious effects of inflammatory burdens on omentin-1 gene expression; so an elevation of omentin-1 serum level after metformin pharmacotherapy may be regarded as potential factor in prevention of diabetic cardiovascular complications [36, 37]; these facts explained the lower atherogenic index (AI) in metformin treated patients that presented with acute MI in the present study.

Additionally, metformin improves omentin-1 serum level even in normal healthy subjects [38]; from herein, metformin treatment even in nondiabetic patients produced a significant cardioprotection directly or indirectly through elevation of protective omentin-1 adipokine [39].

## 5. Conclusion

Metformin pharmacotherapy increases omentin-1 serum level and may be regarded as a potential agent in the prevention of the occurrences of acute MI in diabetic patients.

## Figures and Tables

**Table 1:** Clinical and metabolic characteristics of the patients with type II DM at coronary care unit.

Characteristics	Number (%), mean ± SD
Age (years)	57.5 ± 12.02
Male : female ratio	60% male, 40% female
Race, white : black ratio	100 : 0
Onset of chest pain (hrs)	4.42 ± 2.69 (1–7)
Positive history for IHD	70 (82.35)
Hypertension	69 (81.17)
Hyperlipidemia	79 (92.94)
Duration of hospitalization	6 ± 2.27 (4–10)
Troponin I positive	42 (95.45)
Troponin negative	2 (4.54)
Types of MI	
Anterior	39 (45.88)
Anteroseptal	12 (14.11)
Anterolateral	13 (15.29)
Posterior	14 (16.47)
Inferior	7 (6.81)
Complications	12 (14.11)
Death	2 (2.35)
Arrhythmia	7 (8.23)
Shock	1 (1.17)
Acute heart failure	2 (2.35)
Pharmacotherapy	
Antiplatelets	85 (100)
Anticoagulant	55 (64.70)
Anti-ischemic	78 (91.76)
Antidiabetic agents	85 (100)
Metformin	62 (72.94)
Others	23 (27.05)
ACEI	80 (94.11)
Hypolipidemic agents	78 (91.76)
Analgesics	22 (50)
Direct current shock (DC)	6 (70.58)

ACEI: angiotensin converting enzyme inhibitor.

**Table 2:** Biochemical changes and serum omentin-1 level in acute MI in the patients treated with metformin prior to acute MI in comparison with normal healthy control subjects.

Biochemical parameters	Group III (*n* = 30) Mean ± SD	Group I (*n* = 62) Mean ± SD	*t*	95% CI Upper–lower limits	*p*
S. omentin-1 pg/mL	27.13 ± 1.55	25.43 ± 1.2^*∗*^	6.0073	27.7088–26.5512	<0.0001
S. troponin-I pg/mL	21.202 ± 3.483	75.453 ± 5.62^*∗*^	36.4115	21.4395–20.9645	<0.0001
FBG mg/dL	118.96 ± 9.704	137.75 ± 13.139^*∗*^	10.6056	122.5835–15.3365	<0.0001
Total cholesterol mg/dL	140.96 ± 30.091	152.33 ± 16.877^*∗∗*^	−2.0696	152.1962–129.7238	0.0475
TG mg/dL	138.66 ± 10.366	149.34 ± 23.31^*∗*^	−5.6431	142.5307–134.7893	<0.0001
HDL mg/dL	51.23 ± 5.882	54.43 ± 8.355^*∗*^	−2.9798	53.4264–49.0336	0.0058
VLDL mg/dL	27.64 ± 2.082	29.86 ± 4.662^*∗*^	6.0073	27.7088–26.5512	<0.0001
LDL mg/dL	62.08 ± 3.834	68.13 ± 3.937^*∗*^	−66.274	62.2667–61.8933	<0.0001
AI	0.072 ± 0.008	0.078 ± 0.009^*∗*^	−4.1079	0.075–0.069	0.0003
SPB mmHg	123.16 ± 9.042	140.33 ± 8.773^*∗*^	10.4008	126.5363–119.7837	<0.0001
DPB mmHg	75.50 ± 1.643	82.16 ± 8.773^*∗*^	−22.202	76.1135–74.8865	<0.0001

Group I: patients received metformin prior to acute MI. Group III: normal healthy controls. ^*∗*^ *p* < 0.001;^*∗∗*^ *p* < 0.05.

FBG: fasting blood glucose, TG: triglyceride, HDL: high density lipoprotein, VLDL: very low density lipoprotein, LDL: low density lipoprotein, AI: atherogenic index, SPB: systolic blood pressure, and DPB: diastolic blood pressure.

**Table 3:** Biochemical changes and serum omentin-1 levels in acute MI in the patients that were not previously treated with metformin prior to acute MI in comparison with normal healthy control subjects.

Biochemical parameters	Group III (*n* = 30) Mean ± SD	Group II (*n* = 23) Mean ± SD	*t*	95% CI Upper-lower limits	*p*
S. omentin-1 pg/mL	27.13 ± 1.55	23.71 ± 1.161^*∗*^	12.0852	27.7088–26.5512	<0.0001
S. troponin-I pg/mL	21.202 ± 2.636	79.53 ± 3.60^*∗*^	−121.197	22.1863–20.2177	<0.0001
FBG mg/dL	118.96 ± 9.704	138.77 ± 17.467^*∗*^	−11.181	122.5835–115.3365	<0.0001
Total cholesterol mg/dL	140.96 ± 30.091	242.44 ± 12.552^*∗*^	−18.4716	152.1962–129.7238	<0.0001
TG mg/dL	138.66 ± 10.366	246.55 ± 57.607^*∗*^	−57.007	142.530–134.7893	<0.0001
HDL-C mg/dL	51.23 ± 5.882	38.77 ± 4.570^*∗*^	11.6026	53.4264–49.0336	<0.0001
VLDL-C mg/dL	27.64 ± 2.082	49.30 ± 11.524^*∗*^	−56.9821	28.4174–26.8626	<0.0001
LDL-C mg/dL	62.08 ± 3.834	154.37 ± 4.316^*∗*^	−722.133	62.3414–61.8186	<0.0001
AI	0.072 ± 0.008	0.443 ± 0.0084^*∗*^	−254.006	0.075–0.069	<0.0001
SPB mmHg	123.16 ± 9.042	162.33 ± 10.61^*∗*^	−23.727	126.5363–119.7837	<0.0001
DPB mmHg	75.50 ± 11.643	105.16 ± 21.784^*∗*^	−13.953	79.8476–71.1524	<0.0001

Group II: patients have not received metformin prior to acute MI. Group III: normal healthy controls have not received any drugs.

^*∗*^ *p* < 0.0001. FBG: fasting blood glucose, TG: triglyceride, HDL: high density lipoprotein, VLDL: very low density lipoprotein, LDL: low density lipoprotein, AI: atherogenic index, SPB: systolic blood pressure, and DPB: diastolic blood pressure.

**Table 4:** Biochemical changes and serum omentin-1 level in acute MI in the patients that were not previously treated with metformin prior to acute MI in comparison with the patients previously treated with metformin.

Biochemical parameters	Group I (*n* = 62) Mean ± SD	Group II (*n* = 23) Mean ± SD	*t*	95% CI Upper-lower limits	*p*
S. omentin-1 pg/mL	25.43 ± 1.2^*∗*^	23.71 ± 1.161	11.2861	25.7347–25.1253	<0.0001
S. troponin-I pg/mL	75.453 ± 5.62^*∗*^	79.53 ± 3.60	−5.7122	76.8802–74.0258	<0.0001
FBG mg/dL	137.75 ± 13.139	138.77 ± 17.467	−0.6113	141.0867–134.4133	0.5433
Total cholesterol mg/dL	152.63 ± 16.877^*∗*^	242.44 ± 12.552	−41.9011	156.916–148.344	<0.0001
TG mg/dL	149.34 ± 23.31^*∗*^	246.55 ± 57.607	−32.8371	155.2596–143.4204	<0.0001
HDL-C mg/dL	54.24 ± 8.355^*∗*^	38.77 ± 4.570	14.5794	56.3618–52.1182	<0.0001
VLDL-C mg/dL	29.86 ± 4.662^*∗*^	49.30 ± 11.524	−32.8337	31.0439–28.6761	<0.0001
LDL-C mg/dL	68.13 ± 3.937^*∗*^	154.37 ± 4.316	−13581.08	68.1427–68.1173	<0.0001
AI	0.082 ± 0.09^*∗*^	0.443 ± 0.084	−31.5835	0.1049–0.0591	<0.0001
SPB mmHg	140.33 ± 8.773^*∗*^	162.33 ± 10.61	−19.745	142.5579–138.1021	<0.0001
DPB mmHg	82.16 ± 8.773^*∗*^	105.16 ± 21.784	−20.6431	84.3879–79.9321	<0.0001

Group I: patients received metformin prior to acute MI. Group II: patients have not received metformin prior to acute MI.

^*∗*^ *p* < 0.001. FBG: fasting blood glucose, TG: triglyceride, HDL: high density lipoprotein, VLDL: very low density lipoprotein, LDL: low density lipoprotein, AI: atherogenic index, SPB: systolic blood pressure, and DPB: diastolic blood pressure.

**Table 5:** Serum omentin-1 concentration changes between control normal, healthy subjects and the patients with acute MI that either were previously treated with metformin or not.

S. omentin 1	Mean difference	*t*	df	Sig. (two-tailed)	95% CI
Group I	75.453	75.83	61	0.000301	73.423–77.482
Group II	79.530	93.71	22	0.000586	77.739–81.320
Group III	21.202	182.51	29	0.000545	20.964–21.439

Group I: who received metformin prior to acute MI. Group II: who did not receive metformin prior to acute MI. Group III: normal healthy controls.
